# *Schinus terebinthifolius* Raddi (Brazilian pepper) leaves extract: in vitro and in vivo evidence of anti-inflammatory and antioxidant properties

**DOI:** 10.1007/s10787-023-01316-8

**Published:** 2023-08-28

**Authors:** Marcel da Silva Nascimento, Péligris H. dos Santos, Fabiula F. de Abreu, Andrea Y. K. V. Shan, Ricardo G. Amaral, Luciana N. Andrade, Eliana B. Souto, Matheus I. S. Santos, Ariel de Souza Graça, Jesica B. Souza, Joanda P. Raimundo e Silva, Josean F. Tavares, Ana M. de Oliveira e Silva, Cristiane B. Correa, Monalisa M. Montalvão, Sonia Piacente, Cosimo Pizza, Enilton A. Camargo, Charles dos Santos Estevam

**Affiliations:** 1 Graduate Program in Physiological Sciences, Federal University of Sergipe, São Cristóvão, Sergipe 49000-100 Brazil; 2 Department of Physiology, Federal University of Sergipe, São Cristóvão, Sergipe 49000-100 Brazil; 3 UCIBIO—Applied Molecular Biosciences Unit, MEDTECH, Laboratory of Pharmaceutical Technology, Department of Drug Sciences, Faculty of Pharmacy, University of Porto, 4050-313 Porto, Portugal; 4 Associate Laboratory i4HB—Institute for Health and Bioeconomy, Faculty of Pharmacy, University of Porto, 4050-313 Porto, Portugal; 5 Health Sciences Center, Postgraduate Program in Natural and Synthetic Bioactive Products, Universidade Federal da Paraíba (UFPB), João Pessoa, PB 58051-970 Brazil; 6 Department of Nutrition, Federal University of Sergipe, São Cristóvão, Sergipe 49000-100 Brazil; 7 Department of Morphology, Federal University of Sergipe, São Cristóvão, Sergipe 49000-100 Brazil; 8 Department of Pharmacy, University of the Study of Salerno, Via Giovanni Paolo II n. 132, 84084 Fisciano, Salerno Italy

**Keywords:** *Schinus terebinthifolius*, Oxidative stress, Phenolic compounds, Inflammation, Cytokines, Medicinal plant

## Abstract

The aim of this work was to evaluate the anti-inflammatory and antioxidant effects of ethyl acetate extract obtained from the leaves of Brazilian peppertree *Schinus terebinthifolius* Raddi (EAELSt). Total phenols and flavonoids, chemical constituents, in vitro antioxidant activity (DPPH and lipoperoxidation assays), and cytotoxicity in L929 fibroblasts were determined. In vivo anti-inflammatory and antioxidant properties were evaluated using TPA-induced ear inflammation model in mice. Phenol and flavonoid contents were 19.2 ± 0.4 and 93.8 ± 5.2 of gallic acid or quercetin equivalents/g, respectively. LC–MS analysis identified 43 compounds, of which myricetin-O-pentoside and quercetin-O-rhamnoside were major peaks of chromatogram. Incubation with EAELSt decreased the amount of DPPH radical (EC_50_ of 54.5 ± 2.4 µg/mL) and lipoperoxidation at 200–500 µg/mL. The incubation with EAELSt did not change fibroblast viability up to 100 µg/mL. Topical treatment with EAELSt significantly reduced edema and myeloperoxidase activity at 0.3, 1, and 3 mg/ear when compared to the vehicle-treated group. In addition, EAELSt decreased IL-6 and TNF-α levels and increased IL-10 levels. Besides, it modulated markers of oxidative stress (reduced total hydroperoxides and increased sulfhydryl contents and ferrium reduction potential) and increased the activity of catalase and superoxide dismutase, without altering GPx activity.

## Introduction

Inflammation is defined as immunological, biochemical, and cellular changes in response to molecular patterns associated to pathogens or cell, tissue damage (Rudrapal et al. 2022; Upadhyay and Dixit 2015). The main clinical signs of this response are pain, heat, and redness, which are associated with the development of edema, vasodilation, and leukocyte migration to the injury site. If this process is not controlled, it leads to increased tissue damage, which worsens the loss of tissue function and drives to chronic inflammatory process (Herrero-Cervera et al. 2022).

Cells, such as macrophages and fibroblasts, are activated locally and systemically, inducing the release of mediators in the inflammatory condition (Upadhyay and Dixit 2015). Among these mediators, pro-inflammatory cytokines such as interleukins (IL-6) and the tumor necrosis factor-α (TNF-α) play a key role in the inflammatory response (Hirano 2020). In addition, several inflammatory stimuli, such as the excess of reactive oxygen (ROS) and nitrogen (RNS) species, contribute to the inflammatory process cascade (Tanabe et al. 2022).

During inflammation, there is a greater formation of ROS and RNS. The imbalance between the production of these species and the antioxidant defense mechanisms leads to oxidative stress, which plays an important role in inflammatory conditions (Doktorovova et al. 2014; Gutteridge and Halliwell 2018).

Several drugs are used to treat inflammation, especially, non-steroidal anti-inflammatory drugs and corticosteroids (Juthani et al. 2017). Despite the wide variety of anti-inflammatory drugs on the market, the adverse effects contribute to the continued need for more research to discover isolated molecules or mixtures of compounds such as those presented in medicinal plants that can serve as therapeutic alternatives (Souza et al. 2015).

Derivatives of herbal products are important sources for the discovery of new drugs (Amaral et al. 2020; Matsuo et al. 2011; Santos et al. 2021). For example, polyphenols are a group of metabolites found in parts of plants that have a series of biological activities, such as anti-inflammatory and antioxidant properties (Durazzo et al. 2019; Pimentel-Moral et al. 2018). There is great interest in the search and identification of secondary metabolites, such as polyphenols and other compounds from plant-based natural sources, since they can have valuable therapeutic potential.

One of the promising medicinal plants to treat inflammation is *S. terebinthifolius* Raddi (Anacardiaceae), known as Brazilian pepper tree. In folk medicine, the bark and leaves are used in as infusions and tinctures, to treat bacterial infections (Martínez et al. 1996), or to promote healing, anti-inflammatory and anti-ulcerogenic effect (Martorelli et al. 2011). The leaves can even be used for the green synthesis of silver nanoparticles (de Oliveira et al. 2021). Phytochemical studies of this species have resulted in the isolation of terpenes, monoterpenes, sesquiterpenes, and flavonoids (El-Massry et al. 2009; Matsuo et al. 2011).

A previous study showed that the acetate fraction of *S. terebinthifolius* leaves has anti-allergic activity when administered orally (Cavalher-Machado et al. 2008b). Although this represents consistent evidence of anti-inflammatory effect after oral administration of a *S. terebinthifolius* leaves fraction, there are no detailed reports on the effect of preparations from *S. terebinthifolius* leaves in a model of skin inflammation after topical application. Thus, in this study, we prepared the ethyl acetate extract from the leaves of *S. terebinthifolius* (EAELSt) and tested the effect of this extract in selected in vitro models regarding its antioxidant effects and cytotoxicity activity, and in an in vivo model of skin inflammation and oxidative stress.

## Materials and methods

### Plant material

The leaves of *S. terebinthifolius* were collected in the municipality of São Cristóvão, State of Sergipe, at the coordinates (10º 55′ 14.8″ S, 37º 06′ 11.9″ O) with registration in the National Management System Genetic Heritage (SISGEN) of number A6AC079. A specimen was identified and properly registered in the herbarium of the Federal University of Sergipe (UFS) with voucher 39,748. Leaves were placed in an oven (model MA-037) at 37 °C, with renewal and air circulation for 48 h until complete dehydration and reduced to powder. The powder from the leaves of *S. terebinthifolius* (3 kg) was subjected to extraction with ethyl acetate solvent by Soxhlet apparatus until complete exhaustion of the plant material. After this period, the material was filtered and concentrated on a rotary evaporator (Büchi^®^ R-200, Merck KGaA, Darmstadt, Germany) under reduced pressure at a temperature of 40 ºC, obtaining 295 g of ethyl acetate extract (EAE; 9.83% yield).

### Quantification of total phenolics and flavonoids

Total phenolics content was quantified using the Folin–Ciocalteu method, as described by Sousa et al. (2007) with modifications. An aliquot of EAELSt (100 µL, 1 mg/mL in methanol) was mixed with 6 mL of distilled water and 500 µL of the Folin–Ciocalteu reagent (1 mol/L) and shaken for 1 min. After adding 2 mL of Na_2_CO_3_ (15%), the mixture was shaken for 30 s. The solution was diluted with distilled water to a final volume of 10 mL, incubated for 2 h at 23 ºC, and the absorbance of the sample was measured by a UV–Vis spectrophotometer, model SP22, at 750 nm against a blank consisting of water and the other reagents. Total phenolics was determined by interpolating the absorbance of the samples against a calibration curve using the gallic acid standard (5–30 µg/mL). The results were expressed in mg of gallic acid equivalents per g of extract (mg of GAE/g). All analyses were performed in triplicate.

Total flavonoids content was quantified using the colorimetric method according to Zhishen et al. (1999) with modifications. Aliquots of the EAELSt sample were mixed with 2 mL of distilled water and NaNO_2_ solution (5%, 0.15 mL). After 6 min, AlCl_3_ solution (10%, 0.15 mL) was added and kept for 6 min. Then NaOH solution (4%, 2 mL) and 0.2 mL of distilled water were added until the volume of 5 mL was completed. Subsequently, the solution was kept at rest for 15 min. Total flavonoids was determined according to the quercetin standard curve, measured by spectrophotometer at 510 nm. The results were expressed in mg of quercetin equivalent/g of extract (mg of QE/g). All analyses were performed in triplicate.

### LC–MS/MS analysis

The EAELSt was analyzed by high-performance liquid chromatography (HPLC, Shimadzu, Kyoto, Japan), using an analytical chromatographic column C18 (Kromasil—250 mm × 4.6 mm × 5 μm), coupled to a mass spectrometer (Ion -TrapAmazonX, Bruker), with ionization by electrospray (ESI). The sample was solubilized in methanol (1 mg/mL), with subsequent filtration through polyvinylidene fluoride filters, with a 0.45 μm mesh. The developed chromatographic method used the solvents, methanol (solvent B) of chromatographic grade and ultrapure water type I (Milli-Q^®^), acidified with formic acid (0.1% v/v) (solvent A), with gradient analysis of concentration (5–100% B in 45 min). The injection volume was 10 μL and the flow rate was 0.6 mL/min. In the mass spectrometer, the samples were subjected to sequential fragmentation in MS3. The parameters used were: 4.5 kV capillary, 500 V final plate off set, nebulizer gas at 35 psi, dry gas (N2) with flow rate of 8 mL/ min, and temperature of 300 ºC. The sample was analyzed in the negative ionization mode and the identification of the compounds was based on the data (MS/MS) reported by the literature.

### Antioxidant activity

#### DPPH free radical scavenging assay

The protocol used in this assay was adapted from Cheng et al. (2006), with modifications (Souto et al. 2020a, b). A stock solution of DPPH (2,2-diphenyl-1-picrilhidrazil, 0.208 mmol/L) was prepared in methanol. In triplicates, 100 µL of methanol (blank), gallic acid (standard curve: 1, 2, 3, 4 and 5 µg/mL), and samples were incubated with 100 µL of DPPH solution for 60 min. The absorbances were then read in a UV/Vis microplate spectrophotometer (SynergyMx^®^, Biotek, Bad Friedrichshall, Germany) at 515 nm.

The effective antioxidant concentration required to decrease the initial DPPH radical concentration by 50% (EC_50_) was calculated using % of DPPH reminiscent over 60 min, as opposed to the sample concentrations. The antioxidant concentration necessary to decrease the initial DPPH concentration by 50% (EC_50_) and antioxidant activity index (AAI) were also used for establishing the antioxidant potential of the samples (Scherer and Godoy 2009).

### Lipoperoxidation assay

For the lipoperoxidation, the method of determining substances reactive to thiobarbituric acid was used (Ohkawa et al. 1979), with modifications (Souto et al. 2020a). The rat brain tissue was removed, and tissue homogenates were prepared in phosphate buffer solution (50 mmol/L; pH 7.0, 1:9 m/v. The homogenate was centrifuged at 800 x*g* in a Beckman centrifuge (4 ºC, 15 min) and the supernatant used in the assay. They were added in tubes (100 µL of rat brain homogenate in phosphate buffer 50 mmol/L, pH 7.4) incubated with 50 µL of different concentrations of EAELSt (200, 300, 400, and 500 µg/mL) at 37 ºC during 30 min. Then 350 µL of acetic acid (20%, pH 3.5) and 600 µL of thiobarbituric acid (TBA, 0.36%) were added. Then they were incubated at a temperature of 85 ºC for 1 h. Subsequently, they were cooled on ice and centrifuged at 500 x*g* for 15 min. The absorbance reading was performed at 532 nm. The results were expressed as a percentage of inhibition. Trolox (100 µg/mL) was used as a positive control. All analyses were performed in triplicate.

### In vitro cell viability

This experiment was carried out in a culture of L929 fibroblasts exposed to different concentrations of the EAELSt, using the methylthiazolyl diphenyl tetrazolium bromide (MTT) technique, as previously described by us (Souto et al. 2020a, b). The cells were maintained in culture, seeded in 96-well culture plates (1 × 10^4^ cells/well) and grown in culture medium (DMEM) containing NaHCO_3_ (1.2 g/L), ampicillin (0.025 g/L), streptomycin (0.1 g/L) and 10% fetal bovine serum. Then, they were incubated with different concentrations of EAELSt (25, 50, 75 or 100 μg/mL), solubilized in dimethyl sulfoxide (DMSO, 0.1%) for 24 h at 37 ºC and in an environment containing 5% CO_2_. Cell viability was assessed by adding an MTT solution (0.5 mg/mL in phosphate buffered saline) to the cells, which were then incubated at 37 ºC for 3 h. After removing the MTT, DMSO was added to the plate for 10 min for the solubilization of the crystals of the tetrazolic salt and the absorbance was measured in a UV/Vis microplate spectrophotometer (SynergyMx^®^, Biotek, Bad Friedrichshall, Germany) at 570 nm. The tests were carried out in triplicate in 3–4 independent experiments. The results were expressed in percentage of cell viability based on normalized absorbance values.

### Evaluation of the anti-inflammatory effect

#### Animals

Male Swiss mice (20–30 g) were obtained from the Animal Center of Federal University of Sergipe. Animals were kept at 21–23 °C with free access to feed and water under a 12-h light/dark cycle. All experiments were carried out according to the guidelines of the Brazilian College of Animal Experimentation and the National Institutes of Health and were approved by the Ethics Committee on Animal Use in Research of Federal University of Sergipe (Approval nº 06/2019).

#### Ear inflammation in mice

Ear inflammation was induced by 12-O-tetradecanoylforbol-acetate (TPA) in mice, according to a previous study (De Young et al. 1989), and adapted to our laboratory conditions (Cercato et al. 2021). Initially, the animals (*n* = 5–6/group) were topically treated in the right ear with TPA (1 µg/ear). After 5 min, EAELSt (0.3, 1 or 3 mg/ear), dexamethasone (0.05 mg/ear; positive control) or vehicle (acetone, 20 µL/ear) were also applied to the ears. In the left ear of each animal, the equivalent volume of acetone was administered topically, and each animal served as its own control for the measurement of edema. Euthanasia was performed with inhaled isoflurane 6 h after induction. Then ear sites were cut out circularly with a punch (8 mm of diameter). The mass of the ear sites was measured. The edema values were expressed as the variation (Δ) of the mass (mg) by subtracting the left ear (non-inflamed) mass from the right ear (inflamed) mass.

#### Myeloperoxidase (MPO) activity assay

Ear samples were collected, weighed, cut into small pieces, and kept in test tubes in the presence of phosphate buffer (50 mmol/L, pH 6.0 containing 0.5% hexadecyl-trimethylammonium bromide). Then they were homogenized, and aliquots were centrifuged. The obtained supernatants were subjected to analysis of MPO activity.

In a 96-well plate, supernatants were added to the *o*-dianisidine dihydrochloride solution (0.167 mg/mL, prepared in 50 mmol/mL potassium phosphate buffer containing 0.005% of H_2_O_2_). Changes in absorbance values at 460 nm for a period of 5 min and the results were expressed as units of MPO (UMPO/mg of tissue), considering 1 UMPO as the amount of enzyme that degrades 1 µmol of H_2_O_2_ at 25 ºC, generating an absorbance variation of 0.0113 absorbance units, as previously described by Bradley et al. (1982).

#### Determination of cytokines concentration

The homogenates of the ears of the different experimental groups were used for cytokine quantification. For this purpose, 96-well microplates coated with monoclonal antibodies specific for IL-6, TNF-α or IL-10 were used. The determination was carried out according to the specifications of the manufacturers (Elisa Kits, ThermoFisher Scientifics Inc., Waltham, Massachusetts, USA).

#### Histological analysis of the ears

In an independent experimental set, ears (*n* = 5) treated with the highest dose of EAE (3 mg/ear) or positive control (dexamethasone, 0.05 mg/ear) were used for histological analysis. Subsequently, the tissues were carefully removed, preserved in formaldehyde (10%), and submitted to routine techniques for histological analysis. Slices of 5 µm were stained with hematoxylin and eosin and were viewed and photographed under an optical microscope (Nikon, Tokyo, Japan) with a 20 × magnification. The edema thickness was measured using the ImageJ^®^ program. For this, four representative photographs of each ear were selected and the mean of five measurements of the thickness of the dermis was obtained (Chibli et al. 2014).

#### Determination of oxidative stress biomarkers

Total hydroperoxides were measured in mice ear as previously described by Jiang et al. (1992). The thiol levels were measured as described by Sedlak and Lindsay (1968). To assess the reducing capacity of samples, FRAP method was used (Oyaizu 1986), with minor modifications. The activity of antioxidant enzymes, namely, superoxide dismutase (SOD), catalase (CAT), and glutathione peroxidase (GPx), was determined as described by Doktorovova et al. (2014).

### Statistical analysis

The data were expressed as mean ± standard error of the mean (SEM) for the experimental number indicated in the legends of the figures and were evaluated for normality by the Shapiro–Wilk tests. As there was no impediment to parametric methods, the data were assessed by one-way analysis of variance (ANOVA) followed by the Tukey multiple comparison test. In all these procedures, the statistical program GraphPad Prism (version 7.0) was used. Values with *p* < 0.05 were considered significant.

## Results

### Quantification of total phenol, total flavonoid, and antioxidant activity

**Fig. 1:**
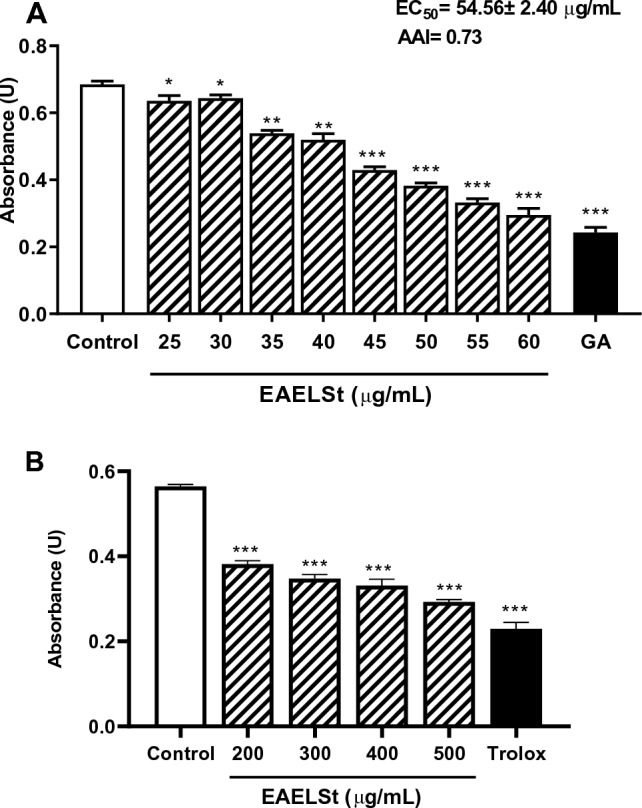
The ethyl acetate extract from leaves of the *S. terebinthifolius* (EAELSt) reduces the amount of 2-diphenyl-1-picryl-hydrazil (DPPH) in vitro. EAELSt was tested against the DPPH radical (**A**) and lipoperoxidation (**B**) in vitro. The results represent men ± SEM of the absorbance values; *n* = 3 experiments in triplicate. Gallic acid (AG; 1 µg/mL) or trolox (100 µg / mL) were used as controls. One-way ANOVA followed by Tukey’s test (*p < 0.05, **p < 0.01 or ***p < 0.001 vs control). *EC*_*50*_ concentration that inhibits 50% of DPPH radical, *AAI* antioxidant activity index

The results show that the total phenol content in EAELSt was 19.21 ± 0.40 mg of GAE/g and the flavonoid content was 93.81 ± 5.17 mg of QE/g. It was also observed that EAELSt reduced the amount of the DDPH radical at concentrations of 25–60 µg/mL compared to the control group (Fig. 1A). Gallic acid (positive control; 1 µg/mL) also significantly reduced the DPPH radical compared to the control. The EC_50_ calculated for the effect of EAELSt by the DPPH method was 54.56 ± 2.40 µg/mL and the IAA was 0.73, which is considered moderate. According to Scherer and Godoy (2009), a poor antioxidant has an AAI < 0.5, while a moderate one has an AAI between 0.5 and 1.0. The strong and very strong antioxidants are defined by AAI values between 1.0 and 2.0, and AAI > 2.0, respectively.

The data in Fig. 1B show that EAELSt significantly reduced spontaneous lipoperoxidation at concentrations of 200–500 µg/mL when compared to the control, which was also observed for the trolox (100 µg/mL).

### LC–MS/MS analysis

**Fig. 2:**
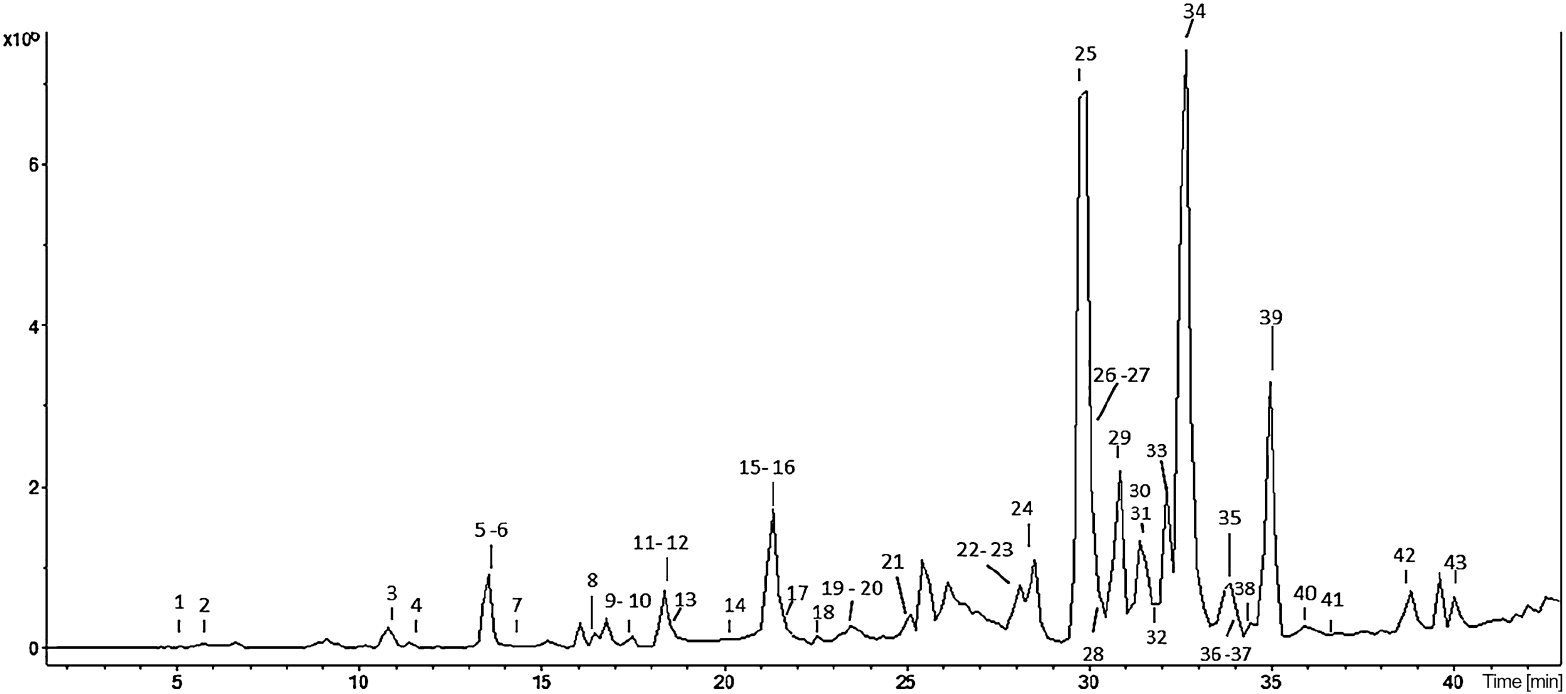
Total chromatogram obtained for the ethyl acetate extract of the leaves of *S. terebinthifolius* by LC–MS/MS

Figure 2 represents the result of the analysis of EAELSt constituents by the LC–MS/MS spectroscopy. Spectroscopic data allowed the identification of 43 substances, for which the identity is detailed in Table 1. The phytochemical profile showed that the EAE is rich in polyphenolic compounds, mostly derived from gallic and ellagic acids. In this table, it is possible to observe that the peaks 25 and 34 have the largest area, which refer to myricetin-O-pentoside and quercetin-O-rhamnoside.

**Table 1:** Identification of constituents of ethyl acetate extract from the leaves of *S. terebinthifolius* (EELSt)

Peak	RT	[M-H]	MS^n^ m/z	Compound
1	5,1	341,0	MS^2^ [341,0]: 178,7; 160,9; 142,8 MS^3^ [341,0 → 178.7]: 160,8; 112.8; 100.7; 88,9	Disaccharide
2	6,6	172,9	MS^2^ [172,9]: 154,8; 110,8; 92,8; 82,9; 72,9	Shikimic acid
3	10,8	330.9	MS^2^ [330.9]: 270.9; 240.8; 210.8; 192.9; 168.8 MS^3^ [330.9 → 168,8]: 124.8	Galloylhexoid
4	12,2	331,0	MS^2^ [331.0]: 270,8; 240.8; 168.9 MS^3^ [331,0 → 270,8]: 210,8; 168,8 MS^3^ [331,0 → 168,9]: 124.6	Galloylhexoid
5	13,2	331,0	MS^2^ [331.0]: 270,8; 240.8; 210,8; 168.8 MS^3^ [331,0 → 168,8]: 124.8	Galloylhexoid
6	13,5	168.8	MS^2^ [168,8]: 124.8; 96,8; 80,9; 68,9	Gallic acid
7	13,9	298,9	MS^2^ [298,9]: 136,8 MS^3^ [298,9 → 136,8]: 92,9	Hydroxybenzoic acid-O-hexoside
8	16,5	325,0	MS^2^ [325,0]: 281,0; 168,8; 154,8; 136,8; 124,8 MS^3^ [325,0 → 168,8]: 124,8	Galloyl-chiquimic acid
9	17,2	483,0	MS^2^ [483,0]: 331,0 MS^3^ [483,0 → 331,0]: 270,8; 210,9; 168,8; 124,9	Digalloyl glucose
10	17,5	325,0	MS^2^ [325,0]: 281,0; 168,8; 124,8 MS^3^ [325,0 → 168,8]: 124,8	Galloyl-chiquimic acid
11	18,0	359,0	MS^2^ [359,0]: 196,9; 181,8; 152,9	Siringenicacid -O-hexoside
12	18,4	320,9	MS^2^ [320,9]: 168,9; 124.8	Digallic acid
13	18,5	152,8	MS^2^ [152,8]: 108,8	Protocatechuic acid
14	20,1	289,0	MS^2^ [289,0]: 244,9; 204.8; 178.8 MS^3^ [289,0 → 244,9]: 226,9; 202,8; 186,8; 160,7	Catechin
15	21,4	320,9	MS^2^ [320,9]: 168,9; 124.8	Digallic acid
16	21,5	182,8	MS^2^ [182,8]: 167,8; 123.8	Methyl gallate
17	21,9	477,0	MS^2^ [477,0]: 324,9 MS^3^ [477,0 → 324,9]: 168,8; 124,8	Digalloylchiquimic acid
18	22,5	473,0	MS^2^ [473,0]: 320,9; 168,8 MS^3^ [473,0 → 320,9]: 168,9; 124.8	Trigallic acid
19	23,4	335,0	MS^2^ [335,0]: 182,8 MS^3^ [335,0 → 182,8]: 167,7; 123,8	Galloylmethyl gallate
20	23,5	472,9	MS^2^ [472,9]: 320,8; 168,7 MS^3^ [472,9 → 320,8]: 168,7; 124.7	Trigallic acid
21	25,0	441,0	MS^2^ [441,0]: 288,9 MS^3^ [441,0 → 288,9]: 244,9; 204,8; 202,9; 178,8; 124,9	Epicatechin-O-gallate
22	25,1	473,0	MS^2^ [473,0]: 320,9 MS^3^ [473,0 → 320,9]: 168,8; 124.7	Trigallic acid
23	28,1	334,9	MS^2^ [334,9]: 182,8 MS^3^ [334,9 → 182,8]: 167,8; 123,8	Galloylmethyl gallate
24	28,5	479,0	MS^2^ [479,0]: 315,9; 316,9; 270,8; 178,9	Myricetin-O-hexoside
25	28,7	449,0	MS^2^ [449,0]: 315,8; 270,8 MS^3^ [449,0 → 315,8]: 270,8; 178,8	Myricetin-O-pentoside
26	29,0	615,0	MS^2^ [615,0]: 462,9; 300,8 MS^3^[615,0 → 300,8]: 270,9; 178,8; 150,9	Quercetin-O-galloyl-hexoside
27	29,7	463,0	MS^2^ [463,0]: 315,9; 270,8; 178,9 MS^3^[463,0 → 315,9]: 286,9; 270,8; 178,8; 150,8; 136,7	Myricetin-O-rhamnoside
28	30,3	615,0	MS^2^ [615,0]: 462,9; 300,9 MS^3^[615,0 → 300,9]: 178,8; 150,8	Quercetin-O-galloyl-hexoside
29	30,8	463,0	MS^2^ [463,0]: 300,9 MS^3^[463,0 → 300,9]: 270,8; 254,8; 178,8; 150,8; 120,8	Quercetin- O-hexoside
30	31,4	433,0	MS^2^ [433,0]: 300,8 MS^3^[433,0 → 300,8]: 270,8; 254,8; 178,8; 150,8; 120,7	Quercetin-O-pentoside
31	31,4	615,0	MS^2^ [615,0]: 462,9; 316,9 MS^3^[615,0 → 316,9]: 270,9; 178,8; 150,8; 136,6	Myricetin-O-(O-galloyl)-deoxyhexoside
32	31,6	585,0	MS^2^ [585,0]: 300,9 MS^3^[585,0 → 300,9]: 178,8; 150,8; 120,8	Quercetin Gallo Pentose
33	32,1	599,0	MS^2^ [599,0]: 312,9; 284,5 MS^3^ [599,0 → 312,9]: 210,7; 168,9; 124,8	Kaempferol-O- (gallo) hexoside
34	32.7	447,0	MS^2^ [447,0]: 300,9 MS^3^ [447,01 → 300,9]: 270,8; 254,9; 178,8; 150,9	Quercetin-O-rhamnoside
35	33,7	417,0	MS^2^ [417,0]: 283,9; 254,9; 226,8 MS^3^ [417,0 → 283,9]: 254,9; 240,9; 226,8	Kaempferol-O-pentoside
36	33,9	599,0	MS^2^ [599,0]: 447,0; 300,9 MS^3^ [599,0 → 300,9]: 178,8; 150,8	Galoilquercetin-O-rhamnoside
37	34,1	585,0	MS^2^ [585,0]: 300,9 MS^3^[585,0 → 300,9]: 178,7; 150,7	Quercetin Gallo Pentose
38	34,6	417,0	MS^2^ [417,0]: 284,9; 254,8; 226,8 MS^3^ [417,0 → 284,9]: 254,8; 241,0; 226,8	Kaempferol-O-pentoside
39	35,0	431,0	MS^2^ [431,0]: 284,9; 254,9; 226,9	Kaempferol-O-rhamnoside
40	36,1	300,9	MS^2^ [300,9]: 273,0; 178,8; 150.8; 120,9	Quercetin
41	36,5	477,0	MS^2^ [477,0]: 314,9; 299,8; 271,8; 242,9	Isorhamnetin-O-hexoside
42	38,8	537,0	MS^2^ [537,0]: 442,9; 416,9; 399,0; 374,9; 330,9	Amentoflavone
43	40,0	327,1	MS^2^ [327,1]: 291,0; 229,0; 210,9; 209,0; 170,8; 164,9	Oxo-dihydroxy-octadecenoic acid

### In vitro cell viability

Table 2 shows that EAE did not alter the viability of L929 fibroblasts at concentrations between 25 and 100 µg/mL when compared to control.

**Table 2:** Effect of the incubation with ethyl acetate extract of the leaves of *S. terebinthifolius* (EAELSt) on L929 fibroblasts viability

Stimulus	Mean of absorbance ± SEM
Control	0.282 ± 0.029
EAELSt (25 µg/mL)	0.267 ± 0.049
EAELSt (50 µg/mL)	0.264 ± 0.012
EAELSt (75 µg/mL)	0.244 ± 0.017
EAELSt (100 µg/mL)	0.250 ± 0.011

Data are shown as mean of absorbance detected at the end of the test. One-way ANOVA followed by Tukey’s test, *n* = 3, experiments performed in triplicate)

### Ear inflammation in mice

**Fig. 3:**
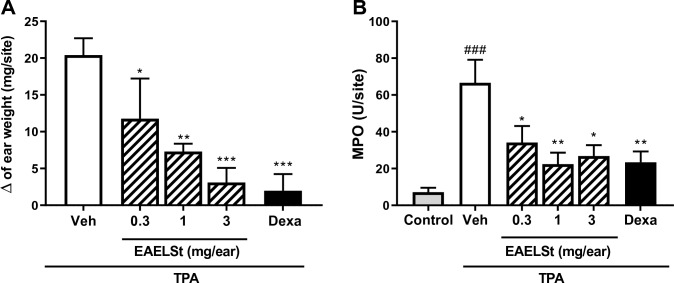
Effect of treatment with ethyl acetate extract from the leaves of *S. terebinthifolius* (EAELSt) on ear edema (**A**) and myeloperoxidase (MPO) activity (**B**) in mice ear. Animals were submitted to concomitant topical administration of 12-O-tetradecanoilforbol-13-acetate (TPA; 1 µg/ear) and EAELSt or dexamethasone (Dexa). Data are shown as mean ± SEM of the variation of ear weight (right–left ear site) and MPO activity (U/ear site) for *n* = 5 animals. One-way ANOVA followed by the Tukey test; ^###^*p* < 0.001 vs. control group (right ear site), *p < 0.05, **p < 0.01 or ***p < 0.001 vs. TPA + vehicle)

The topical application of TPA (1 µg/ear) induced an increase in the mass of mice right ear sites by 20.4 ± 1.0 mg in relation to the left ear, which confirmed the formation of edema (Fig. 3A). This figure also shows that in the ears with administration of EAELSt after TPA, lower ear edema was observed at the doses of 0.3 (*p* < 0.05), 1 (*p* < 0.01), and 3.0 mg/ear of extract (*p* < 0.001) compared with the TPA group. As a positive control, in the ears in which TPA and dexamethasone (0.05 mg/ear) were administered, there was also less edema compared to the TPA plus vehicle group (*p* < 0.001).

Topical application of TPA also increased MPO activity in the animals’ right ear compared to acetone (control) group (*p* < 0.001; Fig. 3B). The activity of this enzyme was lower in the ear of animals submitted to the administration of EAELSt at doses of 0.3 (*p* < 0.05), 1 (*p* < 0.01), and 3.0 mg/ear (*p* < 0.05) compared to the TPA group. Dexamethasone also decreases the MPO activity compared to the TPA plus vehicle group (*p* < 0.01).

#### Cytokine concentration in mice ears

**Fig. 4:**
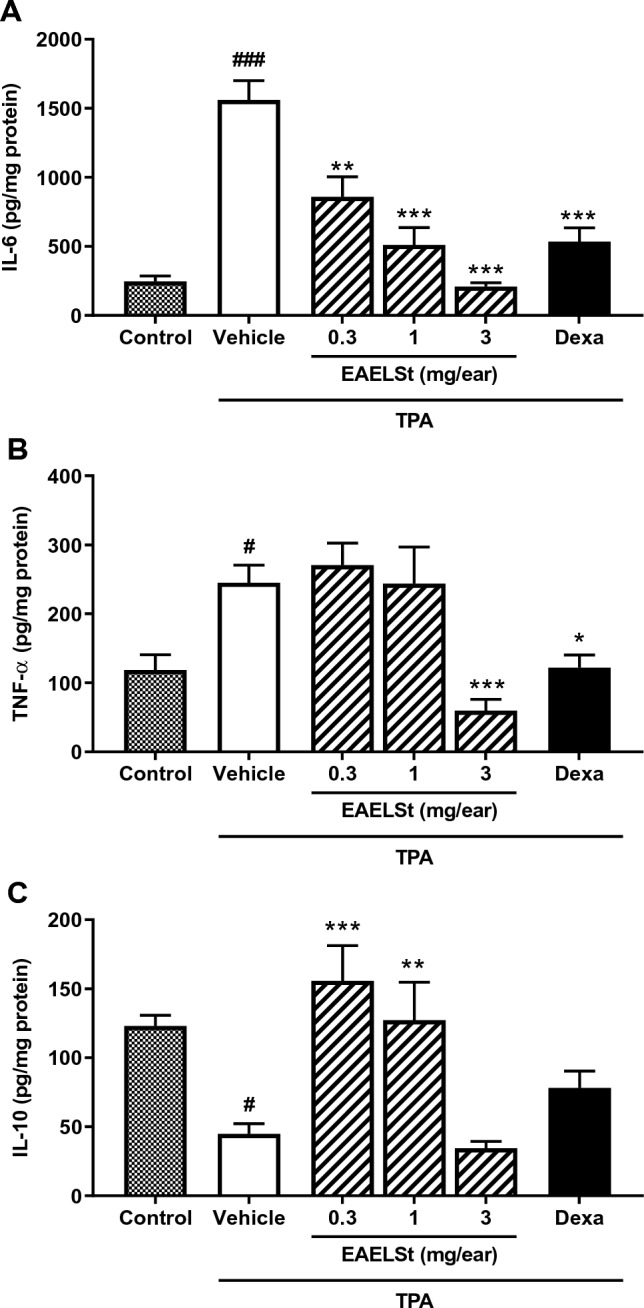
Effect of ethyl acetate extract from the leaves of *S. terebinthifolius* (EAELSt) on cytokines concentration in mice ear. Animals were submitted to concomitant topical administration of 12-O-tetradecanoilforbol-13-acetate (TPA; 1 µg/ear) and EAELSt or dexamethasone (Dexa). IL-6 (**A**), TNF-α (**B**) or IL-10 concentrations are expressed as mean ± S.E.M. (*n* = 4). One-way followed by Tukey test. ^#^*p* < 0.05 or ^###^*p* < 0.001 vs. control group (right ear) and *p < 0.05, **p < 0.01 or ***p < 0.001 vs. TPA + vehicle group

Topical administration of TPA produced a higher concentration of IL-6 and TNF-α in comparison to the acetone group (*p* < 0.001; Fig. 4A, B). The concentration of IL-6 was lower in the ear of animals submitted to administration of EAELSt at doses of 0.3 (*p* < 0.01), 1 (*p* < 0.001), and 3.0 mg/ear (*p* < 0.001) in comparison to TPA group. Only the treatment with EAELSt at 3.0 mg/ear (*p* < 0.001) reduced TNF-α, in comparison to TPA group.

Figure 4C shows that animals submitted to topical administration of TPA presented lower concentration of IL-10 compared to the acetone group (*p* < 0.001). Interestingly, the doses of 0.3 and 1.0 mg/ear reversed the effect of TPA on the IL-10 concentrations (*p* < 0.001 compared to TPA group), leading to values similar to the control group. However, in the group treated with 3.0 mg of EAELSt/ear or dexamethasone (0.05 mg/ear), the concentration of IL-10 was not different from the TPA plus vehicle group.

#### Histological analysis

**Fig. 5:**
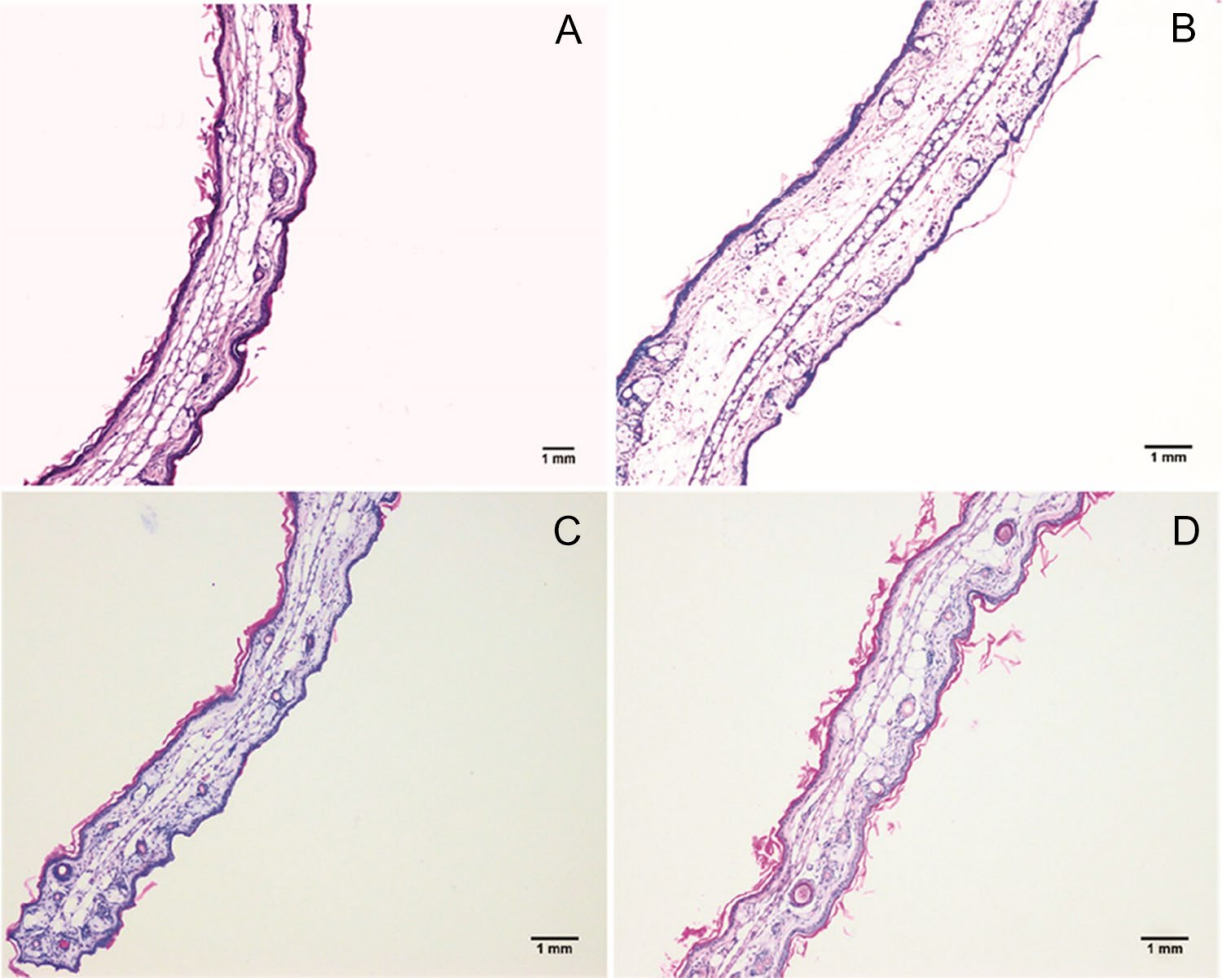
Light microscopic images representative of histological sections of mice ears. Animals were submitted to administration of acetone in the right ear (Control, **A**), 12-O-tetradecanoilforbol-13-acetate (TPA; 1 µg/ear) in the presence of acetone (**B**) or concomitant topical administration of TPA and EAELSt (3 mg/ear) (**C**) or dexamethasone (Dexa, **D**). Scale bars represent 1 mm

Given the effects observed for the inflammatory parameters, the dose of 3 mg/ear was chosen for histological analysis. Representative images from the light microscopy of mice ears are shown in Fig. 5. We observed that the application of TPA (Fig. 5B) increased the ear thickness, with characteristics mainly of edema, that differs from the animal that receives only acetone (control, Fig. 5A). Figure. 5C shows that in the ear submitted to the application of TPA and to the treatment with EAELSt at 3 mg/ear, it was possible to observe the preservation of the tissue through the reduction of edema. The same occurred for the treatment with dexamethasone (0.05 mg/ear, Fig. 5D).

#### Determination of oxidative stress and antioxidant enzymes activity

Administration of TPA increased total hydroperoxides in mice ear (*p* < 0.01) and this effect was reduced by the treatment with EAELSt at 3 mg/ear (*p* < 0.01), but not at 0.3 or 1 mg/ear, when compared to TPA plus vehicle group (Table 3).

**Table 3:** Effect of the treatment with ethyl acetate extract from leaves of *S. terebinthifolius* (EAELSt) on oxidative stress biomarkers and antioxidant enzyme activity in mice submitted to ear inflammation model

Group	TH (mol/L)	SH (µmol/mg of protein)	FRAP (Fe^3+^/Fe^2+^)	CAT (ΔE/min/mg of protein)	SOD (U/mg of protein)	GPx (mUA/mg of protein)
Control	2.76 ± 0.51	2.93 ± 0.30	0.63 ± 0.02	0.073 ± 0.007	7.75 ± 0.41	0.87 ± 0.04
TPA + vehicle	5.79 ± 0.42^##^	1.03 ± 0.21^##^	0.34 ± 0.01^###^	0.026 ± 0.002^##^	1.89 ± 0.15^###^	0.73 ± 0.06
TPA + EAELSt (0.3 mg/ear)	4.89 ± 0.42	2.45 ± 0.45*	0.40 ± 0.01	0.085 ± 0.005***	2.34 ± 0.38	0.88 ± 0.21
TPA + EAELSt (1 mg/ear)	6.31 ± 0.60	1.91 ± 0.16	0.38 ± 0.01	0.074 ± 0.014**	2.15 ± 0.31	0.97 ± 0.17
TPA + EAELSt (3 mg/ear)	2.64 ± 0.12**	2.48 ± 0.38*	0.47 ± 0.02***	0.039 ± 0.009	4.33 ± 0.35***	0.94 ± 0.09

Mice ear submitted to topical administration of 12-O-tetradecanoilforbol-13-acetate (TPA; 1 µg/ear) and concomitant treatment with EAELSt. Data are expressed as mean ± S.E.M. for the quantification of total hydroperoxides (TH), sulfhydryl groups (SH), ferrium reducing potential (FRAP), and catalase (CAT), superoxide dismutase (SOD) and glutathione peroxidase (GPx) activities. One-way ANOVA followed by Tukey test (^##^*p* < 0.01 or ^###^ *p* < 0.001 vs. acetone group; *p < 0.05; **p < 0.01 or ***p < 0.001 vs. TPA + acetone)

When assessing the concentration of SH groups, the animals in the TPA group showed a lower content of these groups when compared to animals in the control group (*p* < 0.01). In animals submitted to administration of EAELSt at 0.3 and 3 mg/ear (*p* < 0.05), but not 1 mg/ear, the concentration of sulfhydryl groups was higher than in TPA plus vehicle group. Besides, in animals administered with TPA, FRAP was decreased when compared to control group (*p* < 0.001). This effect was partially reverted by the administration of EAELSt at 3 mg/ear, but not 0.3 or 1 mg/ear, when compared to TPA plus vehicle group (Table 3).

The activities of SOD, CAT, and GPx were also investigated and are shown in Table 3.

Administration of TPA reduced both CAT (*p* < 0.01) and SOD activities (*p* < 0.001), in comparison to control group. In animals treated with 0.3 and 1 mg of EAELSt/ear, we observed that the activity of CAT was higher in comparison to the TPA + vehicle group (*p* < 0.01 for 0.3 mg/ear and *p* < 0.001 for 1 mg/ear), but this effect did not occur in animals administered with 3.0 mg of extract/ear.

On the other hand, animals that received treatment with EAELSt at 3 mg/ear showed greater SOD activity (*p* < 0.001) when compared to the TPA plus vehicle group. However, this difference was not observed in animals that received 0.3 and 1 mg/ear of EAELSt. GPx activity did not differ among the experimental groups (*p* = 0.7496).

## Discussion

In the present study, we show results about the in vitro antioxidant effect and in vivo anti-inflammatory and antioxidant effect of EAELSt in a model of skin inflammation, which seems to correlate with the composition of the extract.

The chemical characterization of the components presented in the EAELSt showed a high concentration of phenolic compounds and total flavonoids, which may have a greater correlation with pharmacological effects. Similarly, El-Massry et al. (2009) observed the presence of a high concentration of phenolic compounds in the ethanolic extract of the leaves of *S. terebinthifolius*, however, using the maceration technique for extraction.

The analysis of the chemical composition of the EAE by LC–MS/MS confirmed the presence of phenolic compounds and their derivatives with a total of 43 compounds identified. Among them, the major peaks area in the chromatogram were for myricetin-O-pentoside, quercetin-O –rhamnoside, and kaempferol-O-rhamnoside. Rosas et al. (2015), using the hydroalcoholic extract of the leaves of *S. terebinthifolius,* identified the presence of polyphenols such as gallic acid, methyl gallate, and penta-galloyl glucose. These data partially corroborate our findings, since these compounds were also identified in EAE, but to a lesser extent. In a study by Uliana et al. (2016), ferulic and caffeic acids, and quercetin were the major components identified by mass spectroscopy in the extracts.

In this study, it was possible to verify an antioxidant potential by reducing the free radical DPPH. The fact that EAE reduced the amount of this radical in all concentrations tested suggests that the chemical constituents of EAE may act as donors of H^+^ which indicates a mechanism for reducing the DPPH free radical (Floegel et al. 2011; Shahidi and Zhong 2015). In the study by El-Massry et al. (2009), a greater antioxidant activity was observed in the ethanolic extract than in the methanolic or dichloromethane extracts from the leaves of *S. terebinthifolius.* These data corroborate our study, considering that EAE presented a high concentration of total phenols and flavonoids, associated with antioxidant capacity. Flavonoids can act directly or indirectly as antioxidants (Jucá et al. 2020), so that the antioxidant activity is related to the amount of hydroxyl group in its structure (Havsteen 2002).

To complement the evaluation of antioxidant activity in vitro, the evaluation method by inhibiting lipoperoxidation in a biological matrix consisting of rat brain homogenate was used. The results obtained indicate that there was a protective effect for the formation of MDA for all evaluated EAELSt concentrations. MDA is formed during oxidative degeneration as one of the products of free radicals and serves as a marker of lipoperoxidation (Alam et al. 2013). Based on the study by Lesjak et al. (2018), it is possible to suggest that EAELSt effect is related to the presence of phenolic compounds, such as quercetin and its derivatives, which has already been shown to have inhibitory effects on MDA (Lu et al. 2018; Tian et al. 2021).

Before the study in a model of skin inflammation in vivo, a cytotoxicity test with L929 fibroblasts was carried out, to verify whether EAELSt presented any cellular toxicity. Using the MTT test, we showed that EAELSt did not have a cytotoxic effect until the concentration of 100 µg/mL in this cell line. There is no information on the toxicity of *S. terebinthifolius* leaves in in vitro studies. However, data from other authors showed that the ethanolic extract of the bark of this plant did not produce acute or subacute toxicity (45 days of administration) in Wistar rats of both sex, indicating that the oral pretreatment does not cause cytotoxic effect (Lima et al. 2009).

Despite the ethnobotanical suggestions of this species having an anti-inflammatory effect, few studies have investigated its chemical composition and its association with anti-inflammatory and antioxidant activity in vivo*.* For this purpose, the TPA-induced ear inflammation model was used to evaluate the topical anti-inflammatory effect of EAELSt in vivo. The time point of 6 h was chosen because it is the peak time of edema formation, and it was previously reported to show infiltration of neutrophils, according to reference study we used to perform this assay (De Young et al. 1989).

In the present study, it was shown that the topical application of EAELSt reduced the edema caused by TPA in the range doses of 0.3–3 mg/ear. These data indicate that EAE influences the vascular component of the inflammatory response that contributes to edema formation, suggesting a topical anti-inflammatory effect. Accordingly, histological analysis reinforced that treatment with EAELSt at the high dose used reduced edema.

The mechanism for the formation of edema induced by TPA is not completely understood. However, evidence indicates that treatment with TPA activates protein kinase C, promotes release of eicosanoid mediators, such as prostaglandins and leukotrienes, increased expression of cyclooxygenase-2, migration of leukocytes, and increased concentrations of cytokines IL-1β and TNF-α (Carlson et al. 1985; Oliveira et al. 2017), which suggests the involvement of these pathways in the anti-inflammatory effect of EAELSt. In agreement with our data, Fedel-Miyasato et al. (2014) showed that oral treatment with the methanolic extract of the leaves of *S. terebinthifolius* reduced the edema induced by Croton oil in mice ear, similar to that observed in the present study, but these authors did not report any other inflammatory markers in their model.

In addition to the anti-edematogenic effect, a similar effect was observed for doses of EAELSt on MPO activity, which reflects the inhibition of neutrophil migration to the inflamed site. Neutrophil infiltration, characteristic of acute inflammation, was assessed indirectly through the activity of MPO, an enzyme located in neutrophil azurophil granules (Jorch and Kubes 2017). The recruitment of neutrophils occurs through the stimulation of cytokines and chemokines, which, in turn, initiates a series of interactions between different types of leukocytes and endothelial cells (Timmerman et al. 2016).

The study by Rosas et al. (2015) corroborates the reduction in neutrophil migration observed in our study. These authors showed that the oral pretreatment with the hydroethanolic extract obtained from the leaves of *S. terebinthifolius* inhibited the migration of neutrophils in a model of pleurisy induced by zymosan. In the same study, using the zymosan-induced arthritis model, there was also a reduction in joint edema and inhibition of neutrophil migration to the joint.

Our data raised the possibility that components of the EAE may act to reduce the migration of neutrophils. In the study by Rosas et al. (2015), gallic acid, another component found in EAE, reduced the in vitro migration of isolated human neutrophils stimulated with N-formylmethionyl-leucyl-phenylalanine. These data reinforce that the phenolic compounds identified in EAELSt may be responsible for the anti-inflammatory effects shown in the present study.

Increased concentration of IL-6 and TNF-α is associated with cutaneous inflammatory response, as well as with other pro-inflammatory cytokines (Murakawa et al. 2006; Scheller et al. 2011). Accordingly, in the study by Blaser et al. (2016), it was shown that the use of a TNF-α antagonist inhibited both edema and TPA-induced concentrations of TNF-α. We chose to measure TNF-alfa and IL-6 because these are cytokines involved since the initial stage of the inflammatory response. Many other inflammatory mediators might also be reduced by the administration of the extract. However, as we worked with the crude extract, the exact mechanism of action cannot be fully disclosed, provided that a variety of compounds that can contribute to the beneficial effects have been identified.

We showed a reduction of IL-6 and TNF-α in the ears treated with EAELSt, which corroborates our data on the reduction of edema and MPO. It is interesting that all doses of EAELSt reduced the concentration of IL-6, but only the highest dose of this extract decreased the concentration of TNF-α, which suggests differential modulation between these cytokines in the evaluated time point.

Other authors observed that the treatment with hydroethanolic extract of the leaves of *S. terebinthifolius* caused a reduction in the concentration of IL-6 and TNF-α in a model of arthritis induced by zymosan in mice (Rosas et al. 2015), which corroborates the effect observed in our study. It is also interesting that the treatment with the ethyl acetate fraction of the leaves of *S. terebinthifolius* decreased the concentrations of chemokines with a Th2 profile, namely eotaxin and CCL5/RANTES in ovalbumin-induced allergic pleurisy in rats (Cavalher-Machado et al. 2008a).

Considering the compounds presented in EAELSt, it is well described that flavonoids can reduce the formation of pro-inflammatory cytokines (Maleki et al. 2019). Thus, it is possible that the anti-edematogenic effect and the reduction in the concentrations of IL-6 and TNF-α by EAELSt, in part, are attributed to the presence of phenolic compounds in this extract, such as quercetin, which can act solely or synergistically, most likely by modulating intracellular signaling pathways such as phosphatidylinositol-3-kinase or other tyrosine kinase proteins (Lolli et al. 2012; Yokoyama et al. 2015) or transcription factors like the nuclear factor κB (Peng et al. 2018).

In the present study, the effect of EAELSt on IL-10 concentrations was also evaluated. The pretreatment with EAELSt only in the lowest doses (0.3 and 1.0 mg/ear) prevented the reduction of IL-10 levels produced by TPA. IL-10 is a cytokine that plays an important role in maintaining homeostasis and in responding to inflammatory stimuli by suppressing pro-inflammatory cytokines (Ouyang and O’Garra 2019). Considering this fact, it is possible to speculate that the concentrations of IL-10 in the ears would be linked to the concentrations of TNF-α. Thus, at the lowest doses of EAELSt, the increased concentrations of IL-10 would be compensating for the lack of reduction in TNF-α concentrations, which did not occur for the highest dose of the extract. Anyway, the results obtained indicate that the treatment with EAELSt modulated this anti-inflammatory cytokine, which confirms the action of this extract in the cutaneous inflammatory response induced by TPA.

These protective actions of EAELSt may be related to the compounds presented in this extract. These phenolic compounds, in addition to being able to modulate signaling pathways and transcription factors (Lolli et al. 2012; Peng et al. 2018; Yokoyama et al. 2015), are known for their antioxidant effects, which could contribute to the action on the inflammatory response. Thus, we also investigated whether EAELSt could alter the oxidative stress that accompanies the induction of skin inflammation induced by TPA.

In fact, treatment with EAELSt promoted modulation of the formation of hydroperoxides, sulfhydryl groups, and the potential to reduce iron. Our data indicate that there was an inhibitory effect on oxidative stress markers (by reducing total hydroperoxides and by increasing the sulfhydryl groups). The formation of hydroperoxides denotes initial stages of lipid peroxidation, since these species are primary products of lipoperoxidation (Esterbauer 1993). In turn, it is known that the sulfhydryl groups are present in the constitution of several proteins and oxidative stress causes oxidation in these groups, resulting in malfunction of the cellular structures (Santos et al. 2011). Thus, it is most likely that the phenolic compounds in EAELSt reduced the formation of hydroperoxides and preserved the sulfhydryl groups from possible changes induced by oxidative stress induced by TPA. Taken together, these parameters show the decrease in the lipid peroxidation, and increase in sulfhydryl groups and in the Fe^2+^/Fe^3+^ rate, which strongly indicates the antioxidant effect of the extract in mice ears.

Additionally, it was observed that the highest dose of EAE increased the reduction potential indicating antioxidant effect through the FRAP method in vivo. It is known that, during oxidative stress, Fe^3+^ reacts with O_2_^−^ becoming Fe^2+^. This occurs through the Fenton reaction, which leads to the formation of hydroxyl radical which is highly reactive (Shahidi and Zhong 2015). The data found suggest that the reducing potential of EAELSt possibly occurs by the action of polyphenolic compounds identified in this plant, as proven in other studies (Jeyadevi et al. 2013). In fact, the antioxidant activity of polyphenolic compounds such as quercetin identified in EAELSt is directly related to the amount of hydroxyl group, position, and glycosylation (Cai et al. 2006).

We also found that treatment with this extract increased the activity of SOD activity at 3.0 mg/ear. Since SOD is responsible for the conversion of ·O_2_^−^ to H_2_O_2_ and water, this data shows that there was modulation of this enzyme to protect the tissue against oxidative stress. It has been documented in the literature that SOD can contribute to the resolution of inflammation through apoptosis of neutrophils, regulated by the H_2_O_2_ (Yasui and Baba 2006). For the CAT enzyme, treatment with 0.3 and 1 mg EAE/ear increased the activity of this antioxidant enzyme. CAT catalyzes the conversion of H_2_O_2_ into H_2_O, which indicates that the increased activity of this enzyme results in the detoxification of free radicals.

Another interesting finding was related to the effect induced by the dose of 3 mg EAE/ear, which, despite not modulating CAT activity, reduced the formation of hydroperoxides, preserved the sulphidryl groups and increased SOD activity. One possibility would be that the GPx activity acts in a compensatory way at the different doses of the EAE; however, the GPx activity remained unchanged in all the doses evaluated. In this context, it can be suggested that, at the different doses of EAE, there was a compensatory effect between the activities of CAT and SOD that acted primarily in the detoxification process.

In the literature, studies involving oxidative stress in the model of ear edema used in the present study are still seldomly described. To our knowledge, this is the first study to demonstrate the effect of *S. terebinthifolius* on antioxidant markers and enzymes. It is important to highlight the involvement of oxidative stress in the inflammatory process, as tissue damage during this situation leads to an excess of oxygen and nitrogen reactive species (Hussain et al. 2016) and several transcription factors involved in inflammation, such as the nuclear factor-κB, are activated by ROS (Li et al. 2002). Thus, it is plausible to suggest that the anti-inflammatory effect of EAELSt is associated, in part, with protection against oxidative damage. Besides, it is also possible to assume that the anti-inflammatory effect is not solely due to the antioxidant activity, since the anti-inflammatory effect was detected even when using a lower dose, when the antioxidant effect was not fully achieved. Despite these facts, our data suggest that the EAELSt can be promising in the search for alternatives for the treatment of inflammatory conditions for topical use. Furthermore, this study showed that EAELSt is promising for the treatment of skin inflammation. Finally, this study can serve as a basis for future studies to better understand the pharmacological action and its possible mechanisms of action.

## Conclusion

In this study, it was demonstrated that EAELSt promotes a topical anti-inflammatory effect in an animal model of acute TPA-induced skin inflammation. In addition, this extract showed antioxidant activity both in vitro and in vivo*.* From these data, it can be evidenced that the biological effect presented by EAE can bring perspectives to explore the therapeutic potential of this plant and enable the treatment of inflammatory conditions.

## Data Availability

Enquiries about data availability should be directed to the authors.

## References

[CR1] Alam MN, Bristi NJ, Rafiquzzaman M (2013). Review on in vivo and in vitro methods evaluation of antioxidant activity. Saudi Pharm J.

[CR2] Amaral RG, Gomes SVF, Andrade LN, Dos Santos SA, Severino P, de Albuquerque Junior RLC, Souto EB, Brandao GC, Santos SL, David JM, Carvalho AA (2020). Cytotoxic, antitumor and toxicological profile of passiflora alata leaf extract. Molecules.

[CR3] Blaser H, Dostert C, Mak TW, Brenner D (2016). TNF and ROS crosstalk in inflammation. Trends Cell Biol.

[CR4] Bradley PP, Priebat DA, Christensen RD, Rothstein G (1982). Measurement of cutaneous inflammation: estimation of neutrophil content with an enzyme marker. J Invest Dermatol.

[CR5] Cai Y-Z, Mei S, Jie X, Luo Q, Corke H (2006). Structure–radical scavenging activity relationships of phenolic compounds from traditional Chinese medicinal plants. Life Sci.

[CR6] Carlson RP, O’Neill-Davis L, Chang J, Lewis AJ (1985). Modulation of mouse ear edema by cyclooxygenase and lipoxygenase inhibitors and other pharmacologic agents. Agents Actions.

[CR7] Cavalher-Machado SC, Rosas EC, Brito FDA, Heringe AP, de Oliveira RR, Kaplan MAC, Figueiredo MR, Henriques MDGMDO (2008). The anti-allergic activity of the acetate fraction of *Schinus terebinthifolius* leaves in IgE induced mice paw edema and pleurisy. Int Immunopharmacol.

[CR8] Cavalher-Machado SC, Rosas EC, Brito Fde A, Heringe AP, de Oliveira RR, Kaplan MA, Figueiredo MR, Henriques M (2008). The anti-allergic activity of the acetate fraction of *Schinus terebinthifolius* leaves in IgE induced mice paw edema and pleurisy. Int Immunopharmacol.

[CR9] Cercato LM, Araújo JMD, Oliveira AS, Melo AJO, Lima BS, Dos Santos EWP, Dos S Neto AG, De Albuquerque-Júnior RLC, Duarte MC, Araujo AAS, Silva AMO, Grespan R, Correa CB, Camargo EA (2021). Reduced cutaneous inflammation associated with antioxidant action after topical application of the aqueous extract of Annona muricata leaves. Inflammopharmacology.

[CR10] Cheng Z, Moore J, Yu L (2006). High-throughput relative DPPH radical scavenging capacity assay. J Agric Food Chem.

[CR11] Chibli LA, Rodrigues KC, Gasparetto CM, Pinto NC, Fabri RL, Scio E, Alves MS, Del-Vechio-Vieira G, Sousa OV (2014). Anti-inflammatory effects of Bryophyllum pinnatum (Lam.) Oken ethanol extract in acute and chronic cutaneous inflammation. J Ethnopharmacol.

[CR12] de Oliveira DM, Menezes DB, Andrade LR, Lima FDC, Hollanda L, Zielinska A, Sanchez-Lopez E, Souto EB, Severino P (2021). Silver nanoparticles obtained from Brazilian pepper extracts with synergistic anti-microbial effect: production, characterization, hydrogel formulation, cell viability, and in vitro efficacy. Pharm Dev Technol.

[CR13] De Young LM, Kheifets JB, Ballaron SJ, Young JM (1989). Edema and cell infiltration in the phorbol ester-treated mouse ear are temporally separate and can be differentially modulated by pharmacologic agents. Agents Actions.

[CR14] Doktorovova S, Santos DL, Costa I, Andreani T, Souto EB, Silva AM (2014). Cationic solid lipid nanoparticles interfere with the activity of antioxidant enzymes in hepatocellular carcinoma cells. Int J Pharm.

[CR15] Durazzo A, Lucarini M, Souto EB, Cicala C, Caiazzo E, Izzo AA, Novellino E, Santini A (2019). Polyphenols: a concise overview on the chemistry, occurrence, and human health. Phytother Res.

[CR16] El-Massry KF, El-Ghorab AH, Shaaban HA, Shibamoto T (2009). Chemical compositions and antioxidant/antimicrobial activities of various samples prepared from *Schinus terebinthifolius* leaves cultivated in Egypt. J Agric Food Chem.

[CR17] Esterbauer H (1993). Cytotoxicity and genotoxicity of lipid-oxidation products. Am J Clin Nutr.

[CR18] Fedel-Miyasato LES, Formagio ASN, Auharek SA, Kassuya CAL, Navarro SD, Cunha-Laura AL, Monreal ACD, Vieira MDC, Oliveira RJJG, GMR MR (2014). Antigenotoxic and antimutagenic effects of *Schinus terebinthifolius* Raddi in Allium cepa and swiss mice: a comparative study. Genet Mol Res.

[CR19] Floegel A, Kim D-O, Chung S-J, Koo SI, Chun OK (2011). Comparison of ABTS/DPPH assays to measure antioxidant capacity in popular antioxidant-rich US foods. J Food Compos Anal.

[CR20] Gutteridge JMC, Halliwell B (2018). Mini-review: oxidative stress, redox stress or redox success?. Biochem Biophys Res Commun.

[CR21] Havsteen BH (2002). The biochemistry and medical significance of the flavonoids. Pharmacol Ther.

[CR22] Herrero-Cervera A, Soehnlein O, Kenne E (2022). Neutrophils in chronic inflammatory diseases. Cell Mol Immunol.

[CR23] Hirano T (2020). IL-6 in inflammation, autoimmunity and cancer. Int Immunol.

[CR24] Hussain T, Tan B, Yin Y, Blachier F, Tossou MC, Rahu N (2016). Oxidative stress and inflammation: what polyphenols can do for us?. Oxid Med Cell Longev.

[CR25] Jeyadevi R, Sivasudha T, Rameshkumar A, Ananth DA, Aseervatham GSB, Kumaresan K, Kumar LD, Jagadeeswari S, Renganathan R (2013). Enhancement of anti arthritic effect of quercetin using thioglycolic acid-capped cadmium telluride quantum dots as nanocarrier in adjuvant induced arthritic Wistar rats. Colloids Surf B.

[CR26] Jiang ZY, Hunt JV, Wolff SP (1992). Ferrous ion oxidation in the presence of xylenol orange for detection of lipid hydroperoxide in low density lipoprotein. Anal Biochem.

[CR27] Jorch SK, Kubes P (2017). An emerging role for neutrophil extracellular traps in noninfectious disease. Nat Med.

[CR28] Jucá MM, Cysne Filho FMS, de Almeida JC, Mesquita DDS, Barriga JRDM, Dias KCF, Barbosa TM, Vasconcelos LC, Leal LKAM, Ribeiro JE, Vasconcelos SMM (2020). Flavonoids: biological activities and therapeutic potential. Nat Prod Res.

[CR29] Juthani VV, Clearfield E, Chuck RS (2017). Non-steroidal anti-inflammatory drugs versus corticosteroids for controlling inflammation after uncomplicated cataract surgery. Cochrane Database Syst Rev.

[CR30] Lesjak M, Beara I, Simin N, Pintać D, Majkić T, Bekvalac K, Orčić D, Mimica-Dukić N (2018). Antioxidant and anti-inflammatory activities of quercetin and its derivatives. J Funct Foods.

[CR31] Li JM, Gall NP, Grieve DJ, Chen M, Shah AM (2002). Activation of NADPH oxidase during progression of cardiac hypertrophy to failure. Hypertension (dallas, Tex.:1979).

[CR32] Lima LB, Vasconcelos CFB, Maranhão HML, Leite VR, Ferreira PA, Andrade BA, Araújo EL, Xavier HS, Lafayette SSL, Wanderley AG (2009). Acute and subacute toxicity of *Schinus terebinthifolius* bark extract. J Ethnopharmacol.

[CR33] Lolli G, Cozza G, Mazzorana M, Tibaldi E, Cesaro L, Donella-Deana A, Meggio F, Venerando A, Franchin C, Sarno S, Battistutta R, Pinna LA (2012). Inhibition of protein kinase CK2 by flavonoids and tyrphostins. A structural insight. Biochemistry.

[CR34] Lu N, Sui Y, Tian R, Peng YY (2018). Inhibitive effects of quercetin on myeloperoxidase-dependent hypochlorous acid formation and vascular endothelial injury. J Agric Food Chem.

[CR35] Maleki SJ, Crespo JF, Cabanillas B (2019). Anti-inflammatory effects of flavonoids. Food Chem.

[CR36] Martínez MJ, Betancourt J, Alonso-González N, Jauregui A (1996). Screening of some Cuban medicinal plants for antimicrobial activity. J Ethnopharmacol.

[CR37] Martorelli SBDF, Pinheiro ALB, Souza IAD, Higino JS, Bravo F (2011). Efeito anti-inflamatório e cicatrizante do extrado de hidroalcoólico de *Schinus terebinthifolius* Raddi (Aroeira) a 30% em orabase—Estudo “In vivo”. Int J Dent.

[CR38] Matsuo AL, Figueiredo CR, Arruda DC, Pereira FV, Borin Scutti JA, Massaoka MH, Travassos LR, Sartorelli P, Lago JHG (2011). α-Pinene isolated from *Schinus terebinthifolius* Raddi (Anacardiaceae) induces apoptosis and confers antimetastatic protection in a melanoma model. Biochem Biophys Res Commun.

[CR39] Murakawa M, Yamaoka K, Tanaka Y, Fukuda Y (2006). Involvement of tumor necrosis factor (TNF)-α in phorbol ester 12-O-tetradecanoylphorbol-13-acetate (TPA)-induced skin edema in mice. Biochem Pharmacol.

[CR40] Ohkawa H, Ohishi N, Yagi K (1979). Assay for lipid peroxides in animal tissues by thiobarbituric acid reaction. Anal Biochem.

[CR41] Oliveira AS, Cercato LM, de Santana Souza MT, Melo AJDO, Lima BDS, Duarte MC, Araujo AADS, de Oliveira e Silva AM, Camargo EA (2017). The ethanol extract of *Leonurus sibiricus* L. induces antioxidant, antinociceptive and topical anti-inflammatory effects. J Ethnopharmacol.

[CR42] Ouyang W, O’Garra A (2019). IL-10 family cytokines IL-10 and IL-22: from basic science to clinical translation. Immunity.

[CR43] Oyaizu M (1986). Studies on products of browning reactions: antioxidative activities of product of browning reaction prepared from glucosamine. Jpn J Nutr.

[CR44] Peng H-L, Huang W-C, Cheng S-C, Liou C-J (2018). Fisetin inhibits the generation of inflammatory mediators in interleukin-1β–induced human lung epithelial cells by suppressing the NF-κB and ERK1/2 pathways. Int Immunopharmacol.

[CR45] Pimentel-Moral S, Teixeira MC, Fernandes AR, Arraez-Roman D, Martinez-Ferez A, Segura-Carretero A, Souto EB (2018). Lipid nanocarriers for the loading of polyphenols—a comprehensive review. Adv Colloid Interface Sci.

[CR46] Rosas EC, Correa LB, Pádua TDA, Costa TEMM, Luiz Mazzei J, Heringer AP, Bizarro CA, Kaplan MAC, Figueiredo MR, Henriques MG (2015). Anti-inflammatory effect of *Schinus terebinthifolius* Raddi hydroalcoholic extract on neutrophil migration in zymosan-induced arthritis. J Ethnopharmacol.

[CR47] Rudrapal M, Khairnar SJ, Khan J, Dukhyil AB, Ansari MA, Alomary MN, Alshabrmi FM, Palai S, Deb PK, Devi R (2022). Dietary polyphenols and their role in oxidative stress-induced human diseases: insights into protective, effects antioxidant potentials and mechanism(s) of action. Front Pharmacol.

[CR48] Santos CXC, Anilkumar N, Zhang M, Brewer AC, Shah AM (2011). Redox signaling in cardiac myocytes. Free Radical Biol Med.

[CR49] Santos TS, Santos I, Pereira-Filho RN, Gomes SVF, Lima-Verde IB, Marques MN, Cardoso JC, Severino P, Souto EB, Albuquerque-Junior RLC (2021). Histological evidence of wound healing improvement in rats treated with oral administration of hydroalcoholic extract of vitis labrusca. Curr Issues Mol Biol.

[CR50] Scheller J, Chalaris A, Schmidt-Arras D, Rose-John S (2011). The pro- and anti-inflammatory properties of the cytokine interleukin-6. Biochimica Et Biophysica Acta (BBA) - Mol Cell Res.

[CR51] Scherer R, Godoy HT (2009). Antioxidant activity index (AAI) by the 2,2-diphenyl-1-picrylhydrazyl method. Food Chem.

[CR52] Sedlak J, Lindsay RH (1968). Estimation of total, protein-bound, and nonprotein sulfhydryl groups in tissue with Ellman’s reagent. Anal Biochem.

[CR53] Shahidi F, Zhong Y (2015). Measurement of antioxidant activity. J Funct Foods.

[CR54] Sousa CMDM, Silva HRE, Vieira-Jr GM, Ayres MCC, Costa CLSD, Araújo DS, Cavalcante LCD, Barros EDS, Araújo PBDM, Brandão MS, Chaves MH (2007). Fenóis totais e atividade antioxidante de cinco plantas medicinais. Quim Nova.

[CR55] Souto EB, Souto SB, Zielinska A, Durazzo A, Lucarini M, Santini A, Horbańczuk OK, Atanasov AG, Marques C, Andrade LN, Silva AM, Severino P (2020). Perillaldehyde 1,2-epoxide Loaded SLN-tailored mAb: production, physicochemical characterization and in vitro cytotoxicity profile in MCF-7 cell lines. Pharmaceutics.

[CR56] Souto EB, Zielinska A, Souto SB, Durazzo A, Lucarini M, Santini A, Silva AM, Atanasov AG, Marques C, Andrade LN, Severino P (2020). (+)-limonene 1,2-epoxide-loaded SLNs: evaluation of drug release, antioxidant activity, and cytotoxicity in an HaCaT cell line. Int J Mol Sci.

[CR57] Souza GV, Simas AS, Bastos-Pereira AL, Frois GRA, Ribas JLC, Verdan MH, Kassuya CAL, Stefanello ME, Zampronio AR (2015). Antinociceptive activity of the ethanolic extract, fractions, and aggregatin D isolated from sinningia aggregata tubers. PLoS ONE.

[CR58] Tanabe S, O’Brien J, Tollefsen KE, Kim Y, Chauhan V, Yauk C, Huliganga E, Rudel RA, Kay JE, Helm JS, Beaton D, Filipovska J, Sovadinova I, Garcia-Reyero N, Mally A, Poulsen SS, Delrue N, Fritsche E, Luettich K, La Rocca C, Yepiskoposyan H, Klose J, Danielsen PH, Esterhuizen M, Jacobsen NR, Vogel U, Gant TW, Choi I, FitzGerald R (2022). Reactive oxygen species in the adverse outcome pathway framework: toward creation of harmonized consensus key events. Front Toxicol.

[CR59] Tian R, Jin Z, Zhou L, Zeng XP, Lu N (2021). Quercetin attenuated myeloperoxidase-dependent HOCl generation and endothelial dysfunction in diabetic vasculature. J Agric Food Chem.

[CR60] Timmerman I, Daniel AE, Kroon J, van Buul JD (2016). Leukocytes crossing the endothelium: a matter of communication. Int Rev Cell Mol Biol.

[CR61] Uliana MP, Fronza M, da Silva AG, Vargas TS, de Andrade TU, Scherer R (2016). Composition and biological activity of Brazilian rose pepper (*Schinus terebinthifolius* Raddi) leaves. Ind Crops Prod.

[CR62] Upadhyay S, Dixit M (2015). Role of polyphenols and other phytochemicals on molecular signaling. Oxid Med Cell Longev.

[CR63] Yasui K, Baba A (2006). Therapeutic potential of superoxide dismutase (SOD) for resolution of inflammation. Inflamm Res.

[CR64] Yokoyama T, Kosaka Y, Mizuguchi M (2015). Structural Insight into the Interactions between death-associated protein kinase 1 and natural flavonoids. J Med Chem.

[CR65] Zhishen J, Mengcheng T, Jianming W (1999). The determination of flavonoid contents in mulberry and their scavenging effects on superoxide radicals. Food Chem.

